# A new tool for fitting C_4_ gas exchange response curves

**DOI:** 10.1093/jxb/erag308

**Published:** 2026-09-01

**Authors:** Nicholas G Smith, Monika R Kelley

**Affiliations:** Department of Biological Sciences, Texas Tech University, Lubbock, TX 79409, USA; Department of Biological Sciences, Texas Tech University, Lubbock, TX 79409, USA

**Keywords:** Carboxylation, electron transport, *J*_max_, photosynthesis modeling, *V*_cmax_, *V*_pmax_

## Abstract

This article comments on:

**Woodford R, Ermakova M, Furbank RT, von Caemmerer S.** 2026. Fitting photosynthetic carbon dioxide and irradiance response curves for C_4_ leaves. Journal of Experimental Botany **77**, 4987–4999. https://doi.org/10.1093/jxb/erag223

This article comments on:

**Woodford R, Ermakova M, Furbank RT, von Caemmerer S.** 2026. Fitting photosynthetic carbon dioxide and irradiance response curves for C_4_ leaves. Journal of Experimental Botany **77**, 4987–4999. https://doi.org/10.1093/jxb/erag223

**C_4_ photosynthesis is responsible for nearly 20% of global primary productivity. Thus, understanding and predicting variability in C_4_ photosynthesis across environments and taxa is critical. Woodford *et al*. (2026) present a much-needed tool that enables the use of C_4_ leaf gas exchange data for extracting C_4_ photosynthetic traits. These traits enable better understanding of C_4_ biology and can be integrated into predictive models. Here, we highlight the utility of the tool, the advance it represents, and future directions for C_4_ research. The tool enables improved harmonization of data and models, and will enable better understanding and more reliable and realistic future projections of C_4_ photosynthesis.**

Terrestrial flora can be characterized by the way in which they perform photosynthesis. The two most common groupings are C_3_ and C_4_ plant species. C_3_ species comprise most of the Earth’s plant diversity and productivity. However, C_4_ species, while only comprising ∼5% of all plant species on Earth, cover ∼17% of Earth’s surface and are responsible for ∼20% of global primary productivity (Luo *et al*., 2024). They also include many important agricultural species such as maize and sugarcane, as well as many forage grasses used by livestock. Given the numerous ecosystem services provided by C_4_ species, it is imperative to understand how C_4_ species function under present-day and future projected conditions.

To better understand and predict C_4_ photosynthesis, mathematical models have been developed to describe processes and their responses to environmental conditions. One of the most used and cited models of C_4_ photosynthesis is the von Caemmerer (2000) model, recently updated in 2021 (von Caemmerer, 2021). This model formulates many of the key processes underlying C_4_ photosynthesis, but requires input parameters that may vary. These include parameters related to biochemical capacity and efficiency. The choice of model parameters can result in vastly different estimates of C_4_ photosynthesis (Fig. 1). Additionally, data on these parameters can be useful for understanding variability in leaf and plant strategies across environments and lineages (Monson *et al*., 2025).

**Fig. 1.**
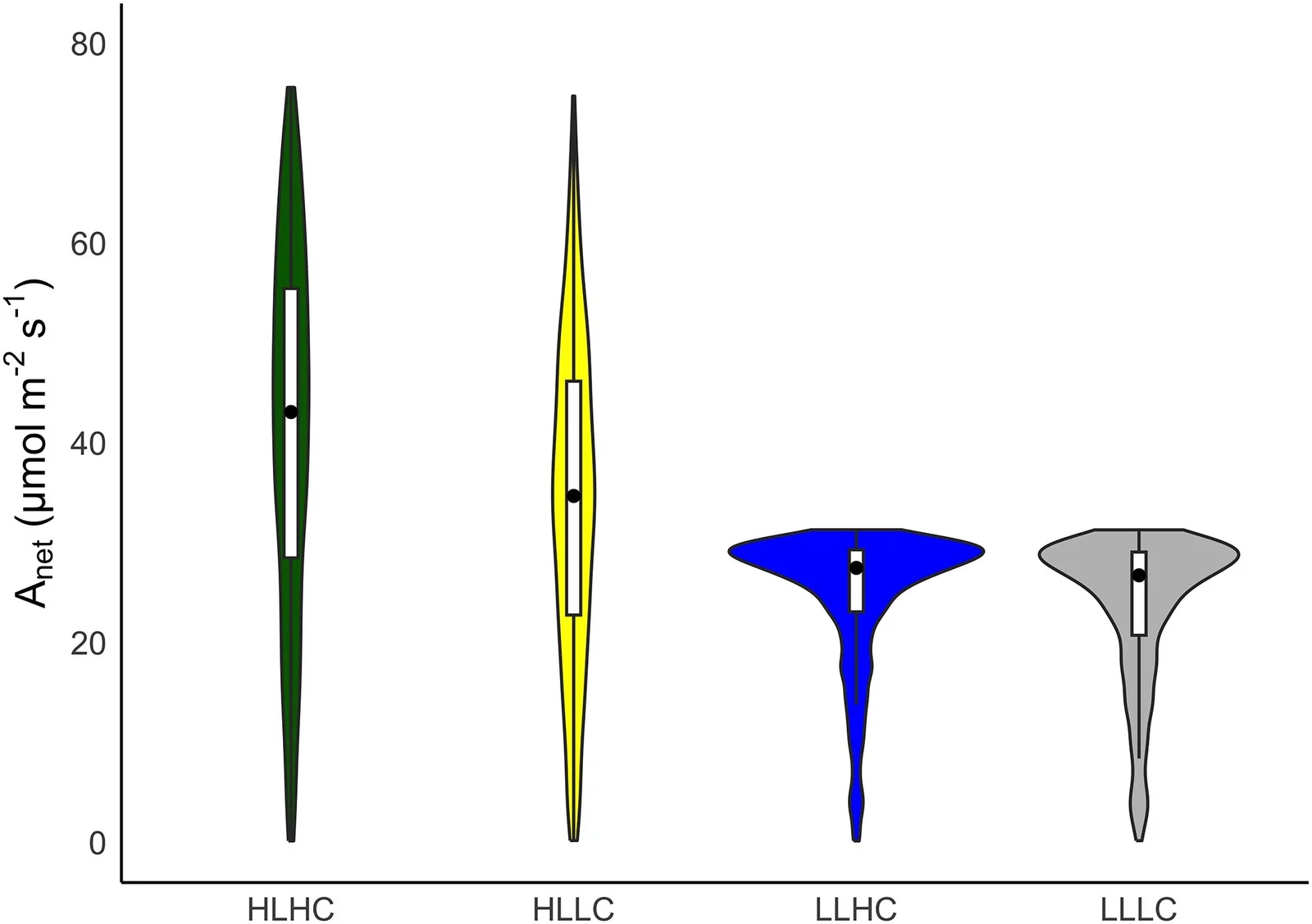
Variation in estimates of C_4_ net photosynthesis resulting from parameter uncertainty. Net photosynthesis (*A*_net_; *y*-axis) under different conditions (*x*-axis) using different biochemical parameters. The conditions represented on the *x*-axis are variation in atmospheric CO_2_ (HC, high CO_2_ = 1000 μmol mol^–1^; LC, low CO_2_ = 150 μmol mol^–1^) and light [HL, high light=1500 μmol m^–2^ s^–1^ photosynthetically active radiation (PAR); LL, low light=400 μmol m^–2^ s^–1^ PAR]. Data were generated using the ‘AciC4’ function from the ‘plantecophys’ package (Duursma, 2015), which implements the von Caemmerer (2000) model of C_4_ photosynthesis. The model was run 1000 times for each of the four conditions by randomly sampling parameter values from normal distributions for each condition. Parameter distributions were generated for the maximum rate of phosphoenolpyruvate carboxylase (PEPc) carboxylation (*V*_pmax_), the maximum rate of Rubisco carboxylation (*V*_cmax_), and the maximum rate of electron transport (*J*_max_). Gaussian normal distributions were generated using the default value from ‘AciC4’ as the mean (*V*_pmax_=120 μmol m^–2^ s^–1^, *V*_cmax_=60 μmol m^–2^ s^–1^, *J*_max_=400 μmol m^–2^ s^–1^) and 50% of that default value as the SD. Violin plots show the distribution of *A*_net_ values from each of the 1000 simulations and are colored by condition (HLHC = green, HLLC = yellow, LLHC = blue, LLLC = gray). Inset boxplots range from the first to third quartile, with whiskers defined as 1.5 times the interquartile range. Black points indicate the median value within a condition.

Leaf gas exchange data can be used to estimate biophysical parameters of C_4_ photosynthesis. However, this requires tools that use mathematical formulations to derive these parameters from measurements. As noted, the mathematical formulations exist, but user-friendly tools for estimating model parameters from gas exchange have lagged behind their equivalents for C_3_ leaves (e.g. Sharkey *et al*., 2007; Duursma, 2015). To fill this gap, Woodford *et al*. (2026) have developed a new Excel tool based on an improved version of the von Caemmerer (2021) model of C_4_ photosynthesis. Below we describe the advance this tool makes over previous data-fitting approaches, highlight how this tool improves our ability to estimate photosynthetic processes in C_4_ leaves, and provide some avenues for improving estimates of photosynthetic processes in C_4_ leaves.

## Current C_4_ fitting tools

Gas exchange response curves for C_3_ leaves are usually fit using the Farquhar, von Caemmerer, and Berry model (Farquhar *et al*., 1980) model of C_3_ photosynthesis, from which fitting tools have been developed (e.g. Sharkey *et al*., 2007; Duursma, 2015). However, there has been a lack of an analogous approach for fitting curves taken on C_4_ leaves. This is due to a variety of factors, including the added complexity of the CO_2_-concentrating mechanisms (CCM) of C_4_ species. This causes multiple limitation states to be indistinguishable from each other. It also causes the necessary addition of processes that may not have defined parameters, such as bundle sheath conductance and leakage of CO_2_, and the flow of electrons through various stages of the photosynthetic processes.

As a result, previous simplified methodologies have been used for C_4_ curve fitting. For CO_2_ response curves, the initial linear portion of the curve at low intercellular CO_2_ has been assumed to be the region where photosynthesis is limited by phosphoenolpyruvate carboxylase (PEPc) carboxylation and used to estimate the maximum rate of PEPc carboxylation (*V*_pmax_). Then, because it is difficult to determine whether Rubisco carboxylation or the electron transport rate for regenerating ribulose-1,5-bisphosphate (RuBP) is limiting photosynthesis, a non-rectangular hyperbola has been used to estimate a maximum rate of photosynthesis (*A*_max_). This has been used in past studies (e.g. Pignon and Long, 2020; Fan *et al*., 2025) to broadly understand environmental and species variation in photosynthetic processes, but does not provide the mechanistic detail needed to quantify the underlying biochemical processes or to parameterize predictive models. Specifically, it does not provide estimates of the maximum rate of Rubisco carboxylation (*V*_cmax_) or the rate of electron transport (*J*). Woodford *et al*. (2026) demonstrate an approach for estimating these parameters that, particularly when combined with light response curves, provides a fuller picture of the photosynthetic strategy used by C_4_ leaves than has been available before.

## Description of this tool and its advances over previous tools

The new gas exchange fitting tool Woodford *et al*. (2026) is an advantage over previous C_4_ parameter estimation methods. First, the tool is grounded in contemporary theory of C_4_ photosynthesis (von Caemmerer, 2021). In fact, the parameterization of cyclic electron flow in this theory has been updated in Woodford *et al*. (2026). Full model equations, parameterizations, and assumptions are provided, allowing users to understand how the tool is getting to the parameter estimates.

Next, the tool provides parameter estimates for both CO_2_ and light response curves, thus delineating what can be gleaned from each type of response curve. This is useful for researchers needing to determine the right protocol for their question. Importantly, Woodford *et al*. (2026) provide a reasonable and cautioned solution for the issue that C_4_ photosynthetic rates at high CO_2_ may be limited by electron transport for regeneration of RuBP or Rubisco carboxylation. That is, CO_2_ response curves use these high CO_2_ data to fit both the *V*_cmax_ and *J* values needed to sustain the measured photosynthetic rates at high CO_2_. The authors suggest using light response curves to estimate the maximum rate of electron transport (*J*_max_) and validate *J* estimates from CO_2_ response curves. Researchers should consider taking both CO_2_ and light response curves to fully estimate model parameters, also argued in Dietze (2014).

**Fig. 2.**
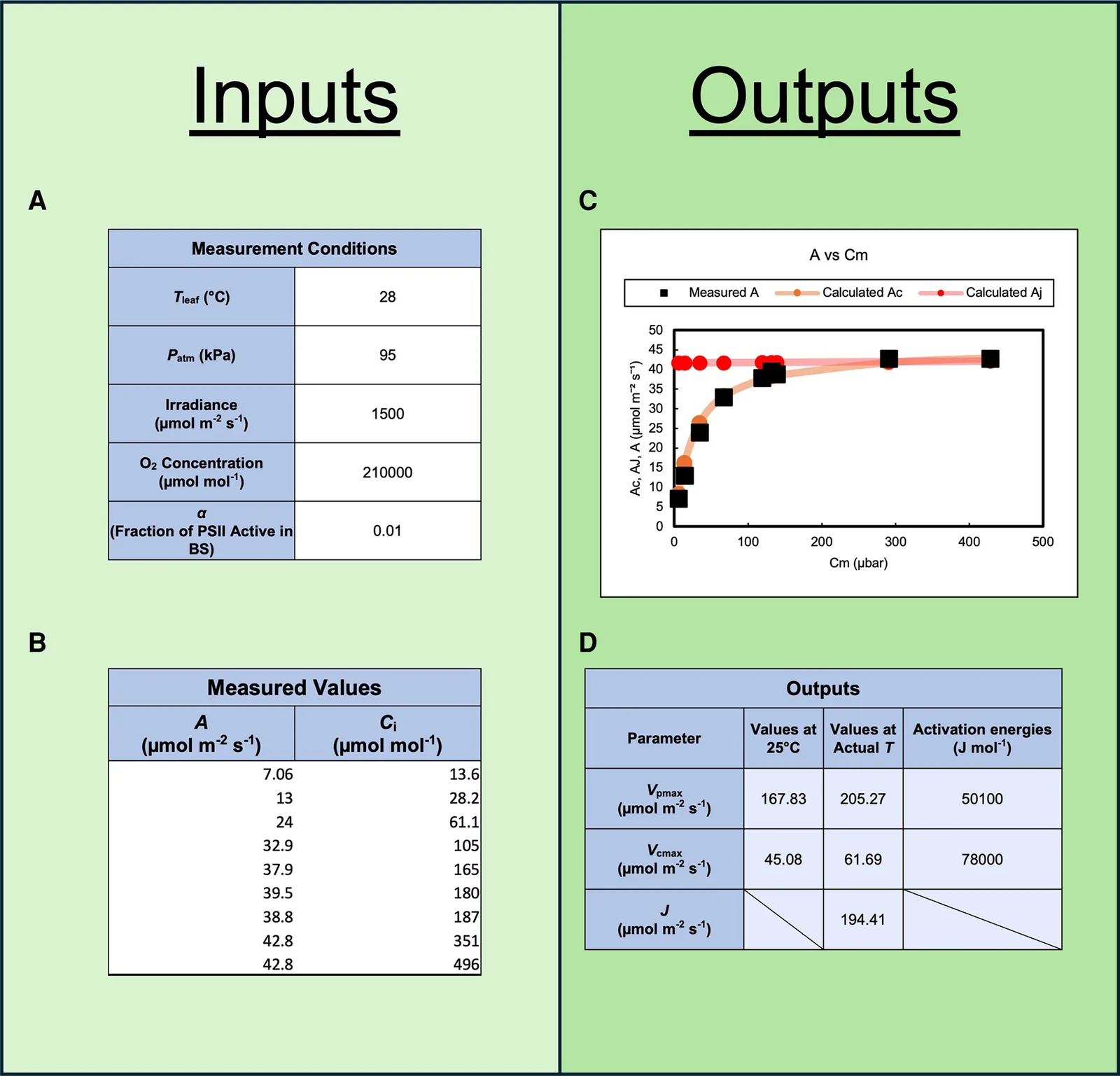
Overview of input (left panels; light green background) and output (right panels; dark green background) windows of the Excel tool developed by Woodford *et al*. (2026) for CO_2_ response curves. (A) Users first define measurement conditions, including leaf temperature (*T*_leaf_), atmospheric pressure (*P*_atm_), irradiance, atmospheric O_2_ concentration, and the fraction of PSII active in the bundle sheath. (B) Users then input measured values of the CO_2_ assimilation rate (*A*) and intercellular CO_2_ (*C*_i_) values obtained from gas exchange measurements. (C) The tool generates a plot of the measured *A* and *C*_i_ values (black squares). Overlaid on top of this are the estimated carboxylation rate-limited photosynthetic values (orange circles) and electron transport rate-limited photosynthetic values (red circles) at each measured *C*_i_ value. These numbers are also provided in a table (table not shown). (D) The tool provides a table with estimated parameter values, including the maximum rate of phosphoenolpyruvate carboxylase (PEPc) carboxylation (*V*_pmax_), the maximum rate of Rubisco carboxylation (*V*_cmax_), and the electron transport rate (*J*). Note that a similar approach is used for fitting light response curves. Data used in this curve-fitting are from *Zea mays* (Smith and Dukes, 2017).

Finally, the tool is presented as an Excel (Microsoft Corporation) document where measured gas exchange values can be entered before estimating model parameters. The tool is simple and easy to use, particularly for those that are familiar with the tool for C_3_ leaves of Sharkey *et al*. (2007). To our knowledge, this is the first such tool for C_4_ leaves. We demonstrate the use of this tool to fit a C_4_ CO_2_ response curve using published data from the publicly available leaf carbon exchange dataset (Smith and Dukes, 2017) in Fig. 2.

## Future directions

The C_4_ gas exchange fitting tool of Woodford *et al*. (2026) is a needed step forward for understanding C_4_ leaf physiology, arriving nearly two decades after the analogous tool for C_3_ leaves (Sharkey *et al*., 2007). However, there is still follow-on work to be done. First, using this tool to develop an open-source, code-based version of the tool would increase accessibility and transparency of the tool—as was shown by Duursma (2015) for C_3_ curves. Next, the tool struggles to ingest high-resolution response curves such as those created from the Dynamic Assimilation Technique (Saathoff and Welles, 2021). As these high-resolution curves become more common, it will be helpful to update the tool to allow a greater number of data points to be input. Additionally, Woodford *et al*. (2026) describe the necessary use of fixed parameters in the model underlying the tool. This should be a call for future research to test the sensitivity of the model to these parameters and design future studies to better quantify these and their variability. Finally, the tool provides an opportunity to harmonize parameters estimated from curves developed in previous studies, for example as was done in Fan *et al*. (2025). This would be helpful for meta-analyses aimed at comparing C_4_ photosynthetic traits across taxa and environments, something that is sorely needed to better understand and predict C_4_ photosynthesis now and into the future.
